# Novel Dental Restorative Solutions for Natural Teeth and Implants

**DOI:** 10.3390/bioengineering9120772

**Published:** 2022-12-06

**Authors:** Gaetano Paolone, Mauro Mandurino, Francesca Pavan, Claudia Mazzitelli, Giuseppe Cantatore

**Affiliations:** 1 Dental School, IRCCS San Raffaele Hospital, Vita-Salute University, 20132 Milan, Italy; 2 Department of Biomedical and Neuromotor Sciences, DIBINEM, Alma Mater University of Bologna, Via S. Vitale 59, 40125 Bologna, Italy

The long-term survival of restorations in the oral cavity has always been one of the most significant challenges in modern dental practice. Despite the continuous evolution of materials technology, dental restorations still fail over time [1,2,3,4,5,6]. In this scenario, preventive dentistry is still considered pivotal to the maintenance of the oral health of patients and preserves the durability of restorations. In daily clinical practice, dentists may face different clinical situations concerning restorative treatments on the teeth or implants. It is, therefore, essential to have a broad knowledge of the applicable therapies, especially in the recent pandemic period, during which educational activities suffered a marked reduction [7]. This collection of articles [8,9,10,11,12,13,14,15,16], published in *Bioengineering,* includes novel restorative solutions for natural teeth and implants. They represent a journey through the different treatment options, relating to different clinical situations. In the first part, conservative treatments, performed with materials that deal with challenging cariogenic biofilm and acidic environments, will be used [8,10,11,12]. In the second part, implant-related solutions will be described [14,15], including a calculation of stress concentration area on mandibular structure [9], novel prosthetic solutions for the appropriate veneering protocol for posterior implant-supported hybrid-abutment restorations [15], or a 3D-printed approach to creating an anatomical cell-seeded scaffold [13]. Nevertheless, among the included articles, one paper focuses on how implant placement is not a risk-free procedure in terms of longevity and proposes a novel medical device to treat these diseases [16]. Despite all the developments in implantology, peri-implant diseases [17], resulting from an improper balance between bacterial load and host defense, are frequent. They can be classified as peri-implant mucositis [18] (an inflammatory lesion of the mucosa surrounding the dental implant) and peri-implantitis [19] (varying from gingivitis to the loss of supporting bone around the implants associated with suppuration and deepened pockets). From this collection of papers, it can be concluded that, despite the continuous evolution of materials and techniques, a conservative approach still appears to be the most suitable option to treat dental diseases.

The oral environment represents a dynamically complex ecosystem in which hard dental tissues are colonized by polymicrobial biofilms, which are the leading cause of caries development [20]. Dental caries is generally associated with an altered oral microflora balance. Several species of bacteria have been associated with caries lesions. The most common are *Streptococcus mutans* (*S. mutans*), *Streptococcus sobrinus*, *Streptococcus mitis* and *Streptococcus oralis* [21], lactobacilli and bifidobacteria [22,23].

*S. mutans* is considered most likely to result in caries’ development; therefore, it is the most-studied microorganism in oral microbiology. *S. mutans*’ virulence is related to its high adhesion capability and acido-genicity on both teeth and dental biomaterials [24,25].

*S. mutans* can produce acids such as lactate and formate as fermentation by-products. *S. mutans* is aciduric or acid-tolerant. It retains its glycolytic abilities at a pH as low as 4.4 [26]. The formation of a biofilm allows for *S. mutans* to survive, whereas other bacteria rarely survive at such a low pH. Dental caries involves the adherence of bacteria and the development of biofilms on both natural and restored tooth surfaces [27]. A biofilm is defined as an aggregate of bacteria in which cells adhere to each other and to a surface [28]. Biofilms are structured microbial communities embedded in a matrix and designed to survive at a solid/fluid interface [29]. It is a complex ecosystem in which bacteria are in equilibrium with the host under health conditions. Any shifts in this equilibrium can lead to dysbiosis [30], and possibly the subsequent development of several localized diseases such as dental caries, periodontal diseases, and other types of infections [28].

Biofilm formation involves four stages [20]:

1. Pellicle formation: tooth or restorative surfaces are covered by organic and inorganic molecules, mainly taken from saliva. These molecules are the basic components of the pellicle and act as receptors for bacteria.

2. Reversible adhesion: single microbial cells or aggregates flow near the solid substrates. In this phase, a weak and reversible physio-chemical interaction occurs between salivary pellicle coating and the microbial surface.

3. Irreversible adhesion: covalent, ionic, or hydrogen bonding is formed. A strong anchorage between bacteria and surface is developed.

4. Co-adhesion and biofilm formation: receptors of firmly attached microorganisms allow for secondary colonizers to adhere, causing biofilm development. A major growth in dental plaque occurs within the biofilm rather than at the surface of the developing biofilm [31], thanks to bacteria’s ability to co-aggregate [32]. Bacteria release several substances in a mature biofilm, such as large amounts of polysaccharides, proteins, nucleic acids, and lipids. These substances concur to maintain biofilm’s structural integrity and provide an ideal matrix for the growth and survival of bacterial cells [33].

The physical and chemical nature of surfaces available in the oral cavity may influence pellicle coating, initial bacterial adhesion and the subsequent biofilm formation. This aspect is highly clinically relevant when it involves restorative materials, especially in the interface, where there is a risk of recurrent (“secondary”) caries. Secondary caries is reported to be the main reason for restoration failures and is frequently related to biofilm formation around dental restorations, especially when performed with resin-based composites (RBC) [5,34]. 

It has been reported that biofilms formed on RBCs differ from those formed on sound dental hard tissues: they are mainly characterized by microorganisms that can survive in a highly acidic environment, such as streptococci and lactobacilli [35,36,37]. Thomas et al. [38] related this behaviour to the fact that RBC surfaces absorb liquids [39] and do not have the buffering effect obtained by the dissolution of calcium ions from the dental mineral matrix. The absence of this buffering effect allows for deeper layers of biofilms to reach lower pH values for an extended period of time. For this reason, natural tissues surrounding a restoration are exposed to a much more intense demineralization challenge.

In order to counteract the formation of biofilm around dental restorations, several clinical strategies may be applied, such as careful finishing and polishing procedures [40,41,42,43] or curing in the absence of oxygen [44]. These procedures may be reduced in terms of time if proper modeling is performed [45,46] with the help of modeling liquids [47].

For this reason, the development of restorative materials that can provide unfavorable conditions for microbial adhesion and colonization represents a promising approach in contemporary dental biomaterials science.

Research and industries are continuously developing RBCs and adhesive systems characterized by prolonged antibacterial agent release or contact-killing surfaces [48,49,50,51,52].

Antimicrobial nanoparticles may, for example, be of particular value [53]. The potential ability of nanoparticles to control oral biofilms is gaining popularity. Thus, the physicochemical characteristics of the nanoparticles mentioned above, such as degree of hydrophobicity, surface charge, and surface-area-to-mass ratio of the plaque biofilm, are being investigated. Incorporating antibacterial compounds inside an RBC may reduce the risk of failure due to secondary caries. The desirable antibacterial effect should preferably be longlasting and should not affect its mechanical properties [54]. 

The antibacterial properties of restorative materials have been investigated since the beginning of the nineteenth century, when it was noted that, when placed in water in contact with copper, Staphylococcus aureus and Lactobacillus acidophilus were killed [55]. Shay et al. were the first to measure the antibacterial properties of dental materials [55].

Nanoparticles may, therefore, be functional because it is easy to set their surface charge [56], hydrophobicity, and other characteristics. Furthermore, nanotherapeutics can be used to control the formation of biofilms as they are characterized by anti-adhesive, delivery, biocidal, and bio-mimetic capabilities [53]. Nanoparticles are, therefore, promising in antibacterial therapies due to their size, high chemical reactivity, and large surface area to mass ratio [57]. Furthermore, being characterized by elevated charge density, they interact with the negatively charged surface of bacterial cells, allowing for antimicrobial activity [58].

The use of nanoparticles and nanotubes to provide antibacterial properties using metal oxides is gaining popularity [12].

Nanoparticles may act in different ways: cell lysis due to the interaction with the peptidoglycan cell wall and membrane; disturbances in protein synthesis; the prevention of DNA replication.

The most commonly used metal oxide nanoparticles are titanium dioxide (TiO_2_), zinc oxide (ZnO), copper oxide (CuO), iron oxide (Fe_2_O_3_), cerium oxide (CeO_2_), tantalum oxide (Ta_2_O_5_), niobium pentoxide (Nb_2_O_5_), zirconium oxide (ZrO_2_) [12].

It is possible to bond drugs to the chemical structure of nanotubes, but they are generally used to carry molecules in their lumen. In dentistry, nanotubes have been used to confer bioactivity or improve physicochemical properties, as well as to carry antimicrobial molecules.

The most commonly used nanotubes are titanium dioxide nanotubes (nt-TiO_2_), halloysite nanotubes (HNT), and boron nitride nanotubes (BNNTs) [12].

Another strategy is the development of dental materials with antibacterial properties using contact-active technology, in which the antimicrobial agent is incorporated inside the matrix of the material and is not supposed to be released [59,60,61]. Surfaces that effectively kill bacteria without releasing a biocide represent a trend towards permanently sterile materials. Nevertheless, this seems very difficult to obtain in the oral environment, while biofilms are known to be able to colonize any surface (natural or artificial) if given enough time. The added value of a contact-active biomaterial is that it could inactivate bacteria directly upon contact, avoiding the release of compounds that might be toxic to mammalian cells as well as bacteria. A contact-active surface can be obtained by co-polymerizing biocidal compounds inside RBCs’ matrix or by the chemical grafting of antimicrobial polymers or carriers to obtain a coating for dental materials.

Although RBCs with contact-killing surfaces and materials featuring the long-term release of antimicrobial agents are currently being developed [50,59], biofilm formation on the surface of conventional RBCs can also be modulated by optimization of their surface properties to obtain surfaces with reduced biofilm development [62]. The conventional wisdom is that a reduction in the surface roughness and surface-free energy of a dental material leads to a decrease in microbial adherence and biofilm formation [63,64].

The oral environment is also characterized by acid attacks that may harm either dental tissues or restorative materials. Perera et al. [10] investigated five glass ionomer cements (GIC) exposed to three acidic solutions, citric acid, phosphoric acid and lactic acid, in order to simulate a highly acidic oral environment. The authors reported a higher acid resistance for new-generation GIC, which are commonly characterized by smaller glass particles. Although these materials have better properties, they are still affected by severe acid attacks (10% citric acid) in citrus-based juices and carbonated beverages [65].

Despite their downsides, some acidic beverages, such as red and white wines, which contain tartaric, citric, succinic, malic, lactic and acetic acids, are probably responsible for antibacterial activity against *S. mutans* [66]. Red and white wines contain polyphenols. Several studies have analyzed the effects of various drinks and foods containing polyphenol groups in decay prevention [67,68]. Polyphenols have also been shown to play an interesting role against oral diseases, infections and cancers [69]. Among their favorable properties, they have antibacterial activity, antioxidant effects and the ability to improve the mechanical and functional properties of biomaterials [70,71]. Tea is another beverage rich in polyphenols [72]. Ferrazzano et al. [73] compared the in vivo effect of tea polyphenol mouthwash and placebo mouthwash against bacteria. They reported a significant lowering of the *S. mutans* (60%) and lactobacilli (42.4%) levels when green tea mouth-rinse was used. Kharouf and colleagues reviewed the existing literature about the dental applications of polyphenols [8]. They reported that epigallocatechin-3-gallate (EGCG), which is a condensed polyphenol, when incorporated in an adhesive resin at concentrations of 0.5% and 1% (*w*/*v*), could enhance the bond strength values and maintain the hybrid layer over time [74,75]. The dental adhesive resin also incorporates toxic particles such as Bis-GMA, TEGDMA and Bis-phenol A [76]. Fonseca et al. [77] reported that the presence of 0.5–1% EGCG in the adhesive could also reduce its release of those particles, hence decreasing toxicity, solubility and water sorption. Additionally, the hydrolysable tannins were reported by Kharouf to benefit dental practice [8]. A successful dental root canal treatment depends on a proper cleaning and filling of the root canal system [29,78,79]. The use of a solution containing 25% (*w*/*v*) of tannic acid as the final irrigation, following hydrogen peroxide and sodium hypochlorite, revealed a smoother and cleaner pulp chamber surface compared to hydrogen peroxide and hypochlorite treatment alone [80]. Another study showed additional benefits of tannic acid, reporting that, when incorporated in polycarboxylate cement, it lowers the activity of collagenase and proteolytic enzymes against dentinal collagen [81]. Future investigations will aim to better comprehend the benefits of polyphenols and, in particular, gallic acid, in dental remineralization processes, as they also seem to be able to interact with phosphates [82,83].

Tooth loss is, unfortunately, a frequent occurrence [84]. Different treatment options are now available to target edentulous regions; however, implants are currently the gold standard solution. Dental implants are, in fact, able to restore oral health to near-normal conditions, giving normal function and phonation and ensuring optimal comfort for the patient without damaging adjacent teeth. Therefore, implant positioning is becoming an increasingly frequent procedure. The growth and improvement of implantology favored the development of a wide variety of techniques and materials for implant-supported rehabilitation. In recent years, new materials and rehabilitation approaches have been proposed. The introduction of high-strength ceramics led to the development of metal-free restorations. These new materials were employed in both natural teeth and implants [85,86]. Among the different dental ceramics that are available, zirconium dioxide is the toughest [87] and is applied as a coping material for both tooth-supported and implant-supported restorations; it is also used successfully as an abutment material [88].

Zirconia can be used in monolithic manufacturing and bi-layered structures, which involve the veneering of a ceramic over the zirconia structure. However, bi-layered structures are subject to chipping of the veneering ceramic and fracture of the coping [89,90]; this suggests that the veneering ceramic–zirconia interface may be the problem.

Multiple techniques to veneer zirconia copings are available, such as the hand-layering technique, in which porcelain is directly applied to the coping by hand. This is, therefore, an operator-dependent approach that requires technical expertise; the pressing technique, which requires the veneer to be pressed on the coping with an ingot and a press furnace; milling technique, involving the separate milling of the coping and veneering structures, which will subsequently be fused together with glass-ceramic [91,92]. The latter makes use of CAD/CAM technology in which human error does not represent a significant factor [93].

It appears that the bond strength of veneering porcelain to zirconia coping is mainly influenced by two factors: the strength of the porcelain [94], and the coefficient of thermal expansion between veneering ceramic and zirconia [95].

As previously stated, zirconia is also used to produce abutments for implant-supported restorations. In this field, the novel hybrid-abutment crown aims to achieve improved aesthetics and a higher strength than traditional implant rehabilitations [96]. The hybrid-abutment crown consists of a hybrid abutment, composed of an all-ceramic abutment and a Ti-Base, and an all-ceramic crown. However, scientific evidence and clinical studies on the concrete clinical applicability of the hybrid-abutment concept and implant-supported single crowns are still scarce [97].

Elshiyab et al. investigated the influence of the veneering technique on the fracture resistance of hybrid-abutment crowns, evaluating the fracture resistance and post-fatigue load of zirconia copings veneered with milling technique versus zirconia copings veneered with the hand-layering technique, and both were cemented onto hybrid-abutments on implants [15]. This study concluded that hybrid-abutment zirconia crowns veneered with milled lithium disilicate represent a reliable restoration option, by virtue of their high fracture resistance. A significantly higher fracture load to failure was attributed to zirconia copings veneered with milling technique, compared to their hand-layered veneer counterparts. However, fatigue testing appears to have no significant effect on the fracture load. This study also reported a high veneer chipping rate regarding hand-layered zirconia copings, which suggests that crowns veneered with this technique are likely to fail due to chipping early during clinical service. It was also concluded in this study that the bond between the zirconia abutment and the Ti-Base that constitute the hybrid-abutment itself is reliable, indicating that it is unlikely to fail during clinical service.

However, more evidence and clinical trials are required to assess the validity of these findings regarding hybrid-abutment veneered zirconia crowns.

Although implantology has undergone significant improvements, peri-implant diseases are still a frequent phenomenon. Peri-implant diseases include two different categories: peri-implant mucositis and peri-implantitis. While peri-implant mucositis is characterized by an inflammatory lesion that involves the mucous tissue surrounding the implant [18], peri-implantitis is additionally associated with bone loss around the implant fixture, frequently leading to suppuration and an increase in pocket depth [19,98]. Data regarding the prevalence of peri-implant diseases are scarce [98]. However, recent studies present the following prevalence data: peri-implantitis occurs in 19.8% of the subjects and 9.3% of the implants, while mucositis affects 29.5% of the subjects and 46.8% of the implants [99]; another study reports peri-implantitis in 17% of the subjects and 11% of the implants [100]. Peri-implantitis represents the main cause of implant failure: the process of bone loss that characterizes peri-implantitis can reach a level that compromises osteointegration, leading to implant failure [101].

Different therapies have been employed to treat peri-implant diseases: the use of either topical or systemic antibiotics and antiseptic solutions; non-surgical treatments, which include laser therapy and the use of ultrasound; surgical treatment, which aims to accomplish an open debridement and decontamination of the infected tissue.

Although many therapeutic approaches to peri-implant diseases are described in the current literature, there is not enough evidence to consider one as a gold standard [102].

The use of lasers to treat both periodontitis and peri-implantitis has increased in recent years. Among the numerous types of available lasers, the erbium: yttrium aluminum garnet (Er:YAG) appears to be the most promising in the scope of non-surgical periodontal therapy, allowing for similar results compared to mechanical debridement [103,104].

The Er:YAG laser is indicated to treat periodontal and peri-implant disease due to its potent bactericidal effect, bacterial toxin inactivation properties [103], and ability to achieve plaque and calculus removal from implant surfaces without causing excessive heating of the surrounding bone tissue [105].

In light of these considerations, it appears important to ascertain if these laser treatments may cause alterations in the titanium implant surfaces. Although some studies suggest that the Er:YAG laser does not cause implant surface alterations with average power settings below 1 W and application times of up to 2 min, it appears that surface alterations may occur with higher average power settings (2 W for 30 s) [106].

The effects of any laser are influenced by different factors, such as water layer thickness, pulse energy, shape, and duration, spot size, target morphology variations [107]. This is of particular importance regarding implants considering the vast range of surface treatments available for titanium implants (sandblasting and acid-etching, machining, titanium plasma spraying, anodic oxidizing). Thus, water irrigation and changes in the delivery system of laser irradiation are the main strategies used to minimize potential alterations of the titanium surface caused by the laser.

Fenelon et al. investigated the effects of conical fiber tips versus conventional laser delivery systems and the influence of irrigation on the interaction between infrared laser irradiation and titanium implant surfaces [14]. In this study, Er:YAG laser and neodymium: yttrium aluminum garnet (Nd:YAG) were compared. The first was the main object of interest, while the latter was employed as a positive control since it causes surface modifications to titanium and other metals. Machined and micro-roughened implant samples were subjected to laser irradiation with various energy settings.

The aforementioned study found that, as expected, Nd:YAG laser with conventional delivery systems induced significant surface modifications at both low and high pulse energies. Such surface alterations proved to be proportional to the delivered energy level. However, the same laser, delivered with conical fiber tips, induced no significant surface modification. However, the Er:YAG laser caused no alteration when used with conical fibre tips, regardless of the pulse energy and, with plane fiber tips on low-pulse-energy settings, produced minimal surface effects at a higher pulse energy. Moreover, it appears that water is effective in reducing surface modifications, as opposed to using both lasers dry.

This study reports that conical fiber tips can avoid alterations in the implant surfaces, suggesting, the necessity for further investigation regarding the clinical effectiveness and applicability of modified tip designs.

As previously stated, a gold-standard treatment for peri-implant disease is currently unavailable, and the success rate of the available treatment options is still unsatisfactory [108]. Hence, alternative therapeutic approaches are being evaluated, such as the electromedical device proposed by Cosoli et al. [16]. This article describes a novel device that encompasses both a therapeutic function, based on electricity and electromagnetic radiation, and a diagnostic function, based on bioimpedance.

Electromagnetic radiation could be successfully employed to treat peri-implantitis considering its effects on biological tissues, such as anti-inflammatory effects [109], antibacterial effects [110], bone formation promotion and bone resorption inhibition [111]. Furthermore, electromagnetic radiation counteracts the main traits that characterize peri-implantitis: bacterial colonization, peri-implant mucous tissue inflammation and peri-implant bone loss.

However, it should be considered that the current diagnostic methodology is heavily influenced by the subjectivity and experience of the clinician, since it relies not only on x-ray examination, but also on observations of gingival swelling and colour, bleeding, the probing depth of peri-implant sites or suppuration [112]. The necessity for a more objective diagnostic procedure could be satisfied using bioimpedance analysis. Bioimpedance appears to be correlated with the pathological or physiological state of biologic tissues [113].

Thus, the device described in the aforementioned study can measure the tissue electrical bioimpedance, which is correlated with the health status of the tissue itself, and administer the appropriate radiofrequency electric current. Measuring bioimpedance in advance not only allows for the therapeutic current to be adjusted according to the conditions of the tissue, but it also allows for the treatment area to be more precisely calibrated, hence avoiding the involvement of healthy surrounding tissues. Therefore, bioimpedance measurement can aid the diagnostic procedure by quantifying inflammation severity and objectively diagnosing the impaired area, allowing for a more selective treatment.

The device, carefully designed on the base of data derived from numerical simulations and subsequently subjected to the EMC pre-compliance tests, is currently being employed in about 50 dental offices, with satisfying results. The success rate is approximately 98% for mucositis and 80% for peri-implantitis, which is quite promising considering the unsatisfactory outcomes of the other available treatment options.

The resolution of peri-implant disease is an infrequent occurrence. Leonhardt et al. reported a 58% success rate of surgical treatment in conjunction with antibiotic therapy [114]. Moreover, osseous defects cannot be replenished with regenerative procedures [98]. However, the non-surgical treatment of peri-implant mucositis seems to successfully alleviate the symptoms, but is unable to solve the inflammatory condition [115].

In addition to the promising application of this new device in the treatment of peri-implant disease, its employment in case of inflammation occurring around natural teeth should also be considered, since the underlying inflammatory mechanism is the same. In the future, this novel device could also be used with artificial intelligence technologies. Moreover, its application field could be expanded in the dentistry purview, or to other anatomical districts.

To place an implant, enough bone must be present. In some cases, when the volume of maxillary or mandibular bone is not sufficient, a bone-regeneration procedure can be performed. Enukashvily et al. conducted a preclinical study to test the effect of fibrin glue implants seeded with dental pulp and stem cells coming from periodontal ligament to repair periodontal bone defects [13]. In this study, the authors created a dental pulp stem cells (DPSC) fibrin-based scaffold with a shape corresponding to the jawbone defect. Cells or cell sheets were layered onto the 3D-printed scaffold surface. The authors reported that molding forms makes it possible to use a wider range of materials that cannot be printed, such as fibrin glue. In addition, they stated that the method of molding forms allows for the introduction of biologically active substances and cells to the scaffold. Given the composition of the cell-seeded fibrin gel and the fact that those cells do not cause the proliferation of alloreactive T-lymphocytes, the authors suggest that the DPSC-seeded scaffold is biocompatible. Moreover, in this study, the cells embedded in fibrin glue retained their viability, immunophenotype and osteogenic differentiation ability and, after implantation in mice, the authors observed an increase in the bone tissue volume in the defect area. Periodontal stem cells and DPSC can restore periodontal bone defects [116] and fibrin glue itself enhances bone tissue repair by mimicking the formation of a blood clot in the defect area [117,118]. The fibrin biodegradation time depends on the gel composition but can range from 3 to 21 days [119]. The authors observed the residual gel clot in histological samples of the site defect 28 days after implantation. However, they suggest that the timing of biodegradation should be extended to allow for full tissue recovery. To protract the scaffold degeneration, the authors suggest adding collagen to the fibrin glue, but further studies need to be carried out.

When choosing the most adequate place to perform fixture insertion in the mandibular bone, the available bone volume is not the only factor that must be taken into account. Cervino et al. [120] stated that it is essential to evaluate the mechanical stress generated by dental prosthesis to reduce the injuries caused by unnecessary efforts and overloads. Rivera and colleagues [9] used a meshless finite-elements method (FEM), to assess the highest stress concentration area generated on the mandibular structure. They compared the efficacy of this software with three conventional software simulations in which mesh generation is required (Ansys, SolidWorks and Inventor). The comparison resulted in similar behaviours when evaluating the highest concentration of tension regions, namely the upper ridge of the mandibular condyle. The percentage of error among each software package was less than 10%. The maximum percentage error of the highest stress concentration was less than 7% when comparing SimSolid software to other FEM software. The authors showed a workflow for complete-mouth implant-supported rehabilitation in patients with a total or partial lack of mandibular structure. This procedure integrates a facial computed tomography (CT) scan in a CAD/CAM image. Once a virtual 3D model was created, it provided a powerful tool for determining the most adequate place to fix the dental retention pin. The irregular anatomy of the mandibular bone causes direct or indirect mastication forces, and the bone material properties strongly affect the strain and stress patterns.

## Data Availability

Not applicable.
